# Automated Knee MR Images Segmentation of Anterior Cruciate Ligament Tears

**DOI:** 10.3390/s22041552

**Published:** 2022-02-17

**Authors:** Mazhar Javed Awan, Mohd Shafry Mohd Rahim, Naomie Salim, Amjad Rehman, Begonya Garcia-Zapirain

**Affiliations:** 1 Faculty of Engineering, School of Computing, Universiti Teknologi Malaysia (UTM), Skudai 81310, Johor, Malaysia; shafry@utm.my (M.S.M.R.); naomie@utm.my (N.S.); 2 Department of Software Engineering, University of Management and Technology, Lahore 54770, Pakistan; 3 Artificial Intelligence and Data Analytics Laboratory, College of Computer and Information Sciences (CCIS), Prince Sultan University, Riyadh 11586, Saudi Arabia; arkhan@psu.edu.sa; 4 eVIDA Lab, University of Deusto, 48007 Bilbao, Spain

**Keywords:** artificial intelligence, osteoarthritis, biomedical images, deep learning, segmentation, U-Net, convolutional neural network, knee bone, knee mask, prediction, ACL MR images

## Abstract

The anterior cruciate ligament (ACL) is one of the main stabilizer parts of the knee. ACL injury leads to causes of osteoarthritis risk. ACL rupture is common in the young athletic population. Accurate segmentation at an early stage can improve the analysis and classification of anterior cruciate ligaments tears. This study automatically segmented the anterior cruciate ligament (ACL) tears from magnetic resonance imaging through deep learning. The knee mask was generated on the original Magnetic Resonance (MR) images to apply a semantic segmentation technique with convolutional neural network architecture U-Net. The proposed segmentation method was measured by accuracy, intersection over union (IoU), dice similarity coefficient (DSC), precision, recall and F1-score of 98.4%, 99.0%, 99.4%, 99.6%, 99.6% and 99.6% on 11451 training images, whereas on the validation images of 3817 was, respectively, 97.7%, 93.8%,96.8%, 96.5%, 97.3% and 96.9%. We also provide dice loss of training and test datasets that have remained 0.005 and 0.031, respectively. The experimental results show that the ACL segmentation on JPEG MRI images with U-Nets achieves accuracy that outperforms the human segmentation. The strategy has promising potential applications in medical image analytics for the segmentation of knee ACL tears for MR images.

## 1. Introduction

A well-functionally healthy knee is essential for mobility. One can understand the intricacies of the movement of the knee by getting familiar with the joints and the bones [1]. The knee is a complex joint of different structures, including bones, tendons, ligaments, and muscles [2]. Ligaments are strong bands of tissues that connect one bone to another. The anterior cruciate ligament (ACL), one of two ligaments that cross in the middle of the knee, connects the thigh bone (femur) to the shinbone (tibia) and helps stabilize your knee joint [3]. The ACL tear ruptured causes ACL injuries, osteoporosis, and osteoarthritis, common among spots players who have to execute start–stop [4]. Osteoporosis (OP) is a condition that leads to weakened bones, causing them to break more efficiently [5]. Osteoarthritis is a prevalent long-term condition affecting hundreds of millions of people worldwide. While it can happen at any age, it most commonly develops after fifty. In osteoarthritis, the cartilage wears down over time, and the joint surface becomes rougher, leading to inflammation. It most commonly affects the knee, hips, hands, feet, and lower back [6,7].

**Figure 1:**
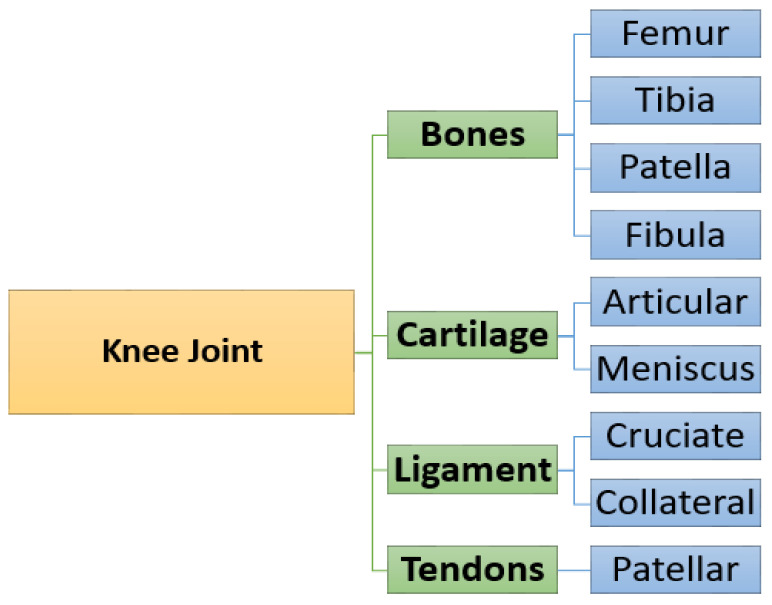
Taxonomy of knee joint anatomy.

The knee joint divides into four main parts, (1) knee bones are involved in the femur, tibia, patella, and fibula. (2) Knee cartilage is the tissue that protects and shields the bones in the joint. (3) Knee ligaments are bands of tough, flexible, fibrous tissues that connect the bones’ ends and hold joints together, and the (4) knee patellar tendon connects the patella kneecap to the tibia [8]. The knee joint anatomy taxonomy [9] is as shown in Figure 1.

**Figure 2:**
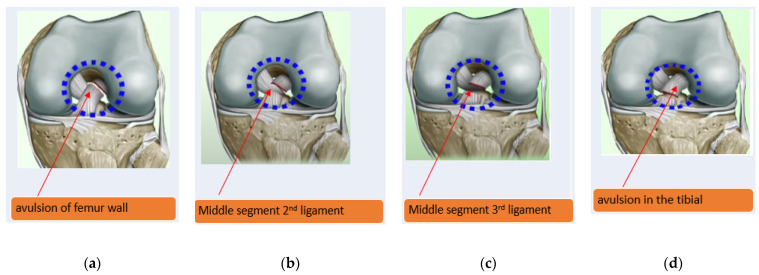
The four types of ACL tears; (**a**) type 1 avulsion of femur wall; (**b**) type II middle segment in 2nd ligament;(**c**) type III middle segment in 3rd of the ligament; and (**d**) type IV avulsion in the tibial part [15].

ACL injuries can lead to developing osteoarthritis of the knee irrespective of treatment. The ACL injuries divide into three grades [10]. The grade I tear is a short-term symptoms sprain or a slight ligament stretch. The grade III tear is a complete rupture that usually requires surgical reconstruction. Grade II is a partial ACL tear or an incomplete tear or injury to the ACL, one of the major ligaments in the knee [11]. The partial ACL injuries occur during sports involving sudden stops or direction changes [12,13]. Four ACL tears in the knee stabilize the knee joint, as shown in Figure 2 [14].

The 3D MRI is a non-invasive and non-ionizing radiation method of studying the soft-tissue structures for ACL tears. It is complicated to detect magnetic resonance imaging (MRI) to determine about the partial tear ligament is damaged. It has very good spatial interpose, submillimeter, and spatial resolution [16].

Image Segmentation is the image processing technique. It provides information about the various regions of interest in the image [17]. It involves classifying each pixel into one or more classes [18,19]. The grouping of pixels together is based on specific characteristics. Image segmentation aims to partition an image into meaningful segments that can be used mainly for medical and other perspectives [20,21]. The elements can often lead to a different type of image segmentation, which can divide into object detection [22] and semantic segmentation. Semantic segmentation refers to the process of linking each pixel [23,24].

An expert radiologist performs the segmentation task manually, but it can be very time-consuming and have variability among radiologists. Moreover, manual delineation is not exactly possible [25]. Segmentation is the critical step for a medical pipeline. It is essential to diagnose, monitor, and treat a patient.

Recently many deep learning approaches have been proposed to solve the problem of segmentation through radiology images in the medical field. The U-Net [26] is a convolutional neural network that was initially designed for semantic segmentation for medical images [27,28].

Although many models have been proposed and implemented, more accurate segmentation approaches are still required for anterior cruciate ligament tears. Therefore, our work aims to present a deep learning framework for MR images mask generation of anterior cruciate ligament tear and segmentation automatically. The modified U-Net was performed results of segmentation above 96% dice coefficient score. The key contributions of this work are abridged in the following key points:

- Make the first effort to prepare pickle MR images into mask and JPEG in the study for segmentation purposes.
- Develop a U-Net CNN architecture after adjusting hyperparameters to ensure the successful segmentation of ACL tears.
- Extensive experiments were performed to calculate scores of accuracy, intersection over union, dice coefficient, precision, recall, F1-score scores and also evaluated through accuracy and dice coefficient loss metrics on training and test values.
- The predicted segment images could classify efficient detection for ACL injury cases.

This paper is organized as follows: In Section 2, recent research related to the segmentation of knee bone diseases regarding MRI is discussed. In Section 3, the dataset and methodology of the proposed U-Net CNN model. In Section 4, some of the experiments result from our segmentation network. The benchmark studies and comparisons with limitations were discussed in Section 5. The last Section 6 conclusion is drawn.

## 2. Research Background

This section delineates several works relevant to our research of semantic segmentation other than ACL, deep learning methods of knee MRI segmentation on various components. The machine learning algorithms were performed better for classification of various diseases instead of segmentation [29,30,31,32,33]. The recent studies have incorporated U-Net CNN architecture for above-knee ACL segmentation.

Prasoon [34] studied segmented knee cartilage with three CNNs on 120,000 training voxels. The method was used 2D features of CNN for each voxel of three planes xy, yz and zx. The performance was measured with dice similarity coefficient, accuracy, sensitivity, and specificity on 114 unseen test data, respectively, 0.8249%, 99.93%, 81.92%, and 99.97%. The dataset of training voxels was limited and did not use the stage layer to the pre-trained CNN model. The advantage of this model was that it trained in a single process of three CNNs.

Deniz et al.’s [35] study was trained on two different architectures; the first was 2D CNN, so-called U-Net [26], that extracted trabecular bone probability map in the proximal femur. The second was 3D CNN into three-dimensional for volumetric segmentation with dilation. The purpose of dilated convolutions was to enlarge the image. The output of the model was performed precision, recall, and dice-similarity score 0.95 after four-fold cross-validation to obtain proximal femur. The AUC value was 0.998 in the case of 2D CNN u-net and 3D CNN with dilated feature maps of 6, 32 with 4 layers. Moreover, the study had institutional review board approval and written informed consent was obtained from 86 subjects of 48 coronal slices for segmentation tasks covering the proximal femur. However, this study was time-consuming, manually segmented by an expert of 86 subjects.

Zhou et al.’s [36] hybrid method was trained on deep CNN based on Convolutional encoder–decoder (CED), conditional random field (CRF), and deformable modeling on musculoskeletal tissue segmentation. CRF aims to identify the contextual relationship among voxels within the same and different classes of tissues. The purpose of deformation was used to refine the output from the 3D CRF. The deep convolutional encoder network pipeline was designed on VGG16 convolutional layers. The encoder network for performing rapid and accurate comprehensive tissue segmentation of the knee joint. The dataset taken from segmentation of knee images 2010 (SK110) image segmentation challenges hosted by the MICCAI [37] training on 19 subjects and 13 classes. The performance was measured by a mean dice coefficient between 0.7 to 0.9. The results performed better in the case of femur and tibia due to the large volume shape. The advantage was less sensitive against the overfitting data. However, the study’s limitations were less accurate than those found in the case of small structures like a meniscus.

Ambellan et al.’s [38] combined approach incorporated statistical shape models (SSMs) [39] with 2D CNN and 3D CNNs to segment femoral bone (FB) and tibial bone (TB) via voting way. The 2D CNN variant of U-Net created segmentation masks of FB and TB, SSMs. Furthermore, fitted on these masks, refinement with 3D CNN to segment MRI taken from SSMs, post-processing SSM to enhance the result of CNN regions on knee FC and TC and the last step applied 3D CNN for segmentation in TC and FC. The time of training was reduced by a factor of 6. The approach was trained on sagittal MRI cartilage subjects of 88 Osteoarthritis Initiative (OAI) Imorphics [40], 507 OAI ZIB [41], and 60 SK110. The accuracy of the model was evaluated on 40 validations and 50 submission datasets of SK110. The DSC scores on the OAI Imorphics baseline dataset performed were 89.4%, 86.1%, 90.4% bones cartilages of femur cartilage, medial tibial cartilage, and lateral tibial cartilage, respectively. The OAI ZIB dataset was shown DSC 98.6% for FB, 98.5% for TB, 89.9% for FC, and 85.6% for TC. However, the total score of DSC has remained 75.73% for SK110 dataset. The processing time will take 43 weeks in a single node when the data set becomes greater than 50,000.

Xu and Niethammer [42] study was used DeepAtlas for jointly deep CNN networks for weakly supervised image registration on unlabeled MR images via an anatomy similarity loss and customized light 3D U-Net. The experimental results were tested on 3D knee MRIs from the Osteoarthritis Initiative (OAI) dataset and 3D brain MRI. The approach was beneficial in case of a lack of manual segmentations. The segmentation of Semi-DeepAtlas DSC was showed 96.80% for bones (femur and tibia) and 77.63% in the case of cartilages (femoral and tibial). The limitation of the study was not investigated multitask.

Burton et al.’s [43] study was trained through 2D, and 3D CNN models for automatic segmentation related to biomechanics orthopedics of 36 labeled MRI a statistical shape model of the knee [44]. The classes for the annotated scan were associated with the femur, femur cartilage, patella, patella cartilage, tibia, and tibial cartilages of 20 males and 16 female subjects. The 51 unlabeled MRIs from the OAI dataset [45] used U-Net architecture.

Liu et al.’s [46] study was applied on cartilage tissues of the femur, tibia, femoral cartilage, and tibial to segment with a 2D convolutional encoder network of a Visual Geometry Group 16 (VGG16) architecture [47,48]. The datasets consisted of 175 patients of MRI cartilage lesion within 17395 image slices. The segmentation results were performed through mean Dice confidence femur, tibia, femoral cartilage, and tibial cartilage, respectively, 0.96, 0.95, 0.81 and 0.82. The average training was taken 20 s for a patient on all image sections with a trained network of cartilage segmentation. However, in the case of the segmentation network, it took 6.2 h in each fold of image data sets. However, one-time training of cartilage classification was a long process. However, the training time of the entire network for diagnostics was rapid. The study has some limitations: the study did not evaluate segmentation and classification on patellar cartilages, the reference standard for presence and absence of articular cartilage was not evaluated by arthroscopy, the study would not be possible to assess through arthroscopy because the exact location of cartilage is not described correctly. The clinical detection system can be applied to the test set for cartilage in the future.

Tack et al.’s [49] study applied convolutional neural network 2D and 3D U-Net in combination with statistical shape models (SSM) [50] of menisci sub-volumes has image the dimensions (384 × 384), (48 × 72 × 16), respectively, on sagittal MRI. These networks were trained on IGS datasets provided by Imorphics on two-fold cross-validation. The segmentation was improved by principal component analysis on three meniscus, lateral, medical, and combined. The segmentation results with dice similarity coefficient were 83.8% for medial menisci (MM) and 88.9%. The study’s limitation was that coronal DESS MRI accuracy did not increase, and cartilage denudation decreased tibia and meniscus extrusion.

Raj’s [51] study proposed a µ-Net based on U-Net 3D-CNN architecture for knee cartilage to segment high resolution 100 3D MR volumes on a MICCAI SKI10 [37] dataset after re-sampled the labels to 128 × 128 × 64 voxels. The model was also validated on the Osteoarthritis Initiative (OAI) datasets [52] of 176 knee 3D MR volumes of 88 patients. The 3D-DESS MR Slice has generated four segmentations by µ-Net femoral cartilage, patellar cartilage, meniscus, and tibial cartilage. The dice score of femoral cartilage and tibial cartilage was 0.834 and 0,825, respectively. The patellar cartilage was a lower score of DSC due to the absence of ground truth.

Pedoia et al.’s [53] study was used 2D U-Net for the cartilage and meniscus segmentation of 11 classes. The exact approach 3D U-Net CNN architecture was used to detect severity staging patellar and meniscal cartilage bounding box in a cascaded manner. The study was taken using 3D FSE CUBE knee MRI of 1481 samples, OA patients of 173 and after anterior cruciate ligament injury 129. The average cropped meniscus volume region was 39 × 79 × 44 voxels out of 5912 meniscus volume of interest. The ROC for binary meniscus lesion of training, validation, and testing was 0.95, 0.84 and 0.89, respectively, whereas in the case of binary lesion, it was 0.99, 0.89, and 0.88 with a ratio of 65, 20, and 15. However, the specificity of meniscus lesion and cartilage was 81.98% and 82.27%, respectively. The limitation of the study was the lack of image annotation uncertainty and the lack of the actual gold standard.

Norman et al. [54] automatic segmentation method was end-to-end, with no extensive pipeline for image registration. The U-Net CNN architecture was applied on 638 MR imaging volumes with a weighted cross-entropy function to handle the class imbalance. The authors used two different datasets, included patients with ACL injuries, patients with OA, (a) the 464 3.0 T MRI T1p-weighted (b) 174 3D double-echo steady-state (DESS) that were taken from OAI. The automatic segmentation performance was calculated by dice coefficient scores for both datasets of six knee MR parts FC, LTC, MTC, PC, LM, and MM. The DSC range remained 0.632 to 0.699 only for the case of T1p-weighted on validation data with a processing time of 2.5 s. In the case of DESS, the DSC range was higher at 0.878 to 0.753 with a lower computational time of 8 s. The study’s limitation was a lack of ground truth, and that it did not segment more parts of the meniscus and cartilages.

Pedoia, Norman, Mehany, Bucknor, Link and Majumdar’s [53] study used 2D U-Net for the cartilage and meniscus segmentation of 11 classes. The same approach 3D U-Net CNN architecture was used to detect severity staging patellar and meniscal cartilage bounding box in a cascaded manner. The study was taken using 3D FSE CUBE knee MRI of 1481 samples, OA patients of 173 and after anterior cruciate ligament injury 129. The average cropped meniscus volume region was 39 × 79 × 44 voxels out of 5912 meniscus volume of interest. The ROC for binary meniscus lesion of training, validation, and testing was 0.95, 0.84 and 0.89, respectively, whereas in the case of binary lesion, it was 0.99, 0.89, and 0.88 with a ratio of 65, 20, and 15. However, the specificity of meniscus lesion and cartilage was 81.98% and 82.27%, respectively. The limitation of the study was the lack of image annotation uncertainty and the lack of an actual gold standard from human interpret images.

Norman, Pedoia and Majumdar’s [54] automatic segmentation method was end-to-end, with no extensive pipeline for image registration. The U-Net CNN architecture was applied on 638 MR imaging volumes with a weighted cross-entropy function to handle the class imbalance. The authors used two different datasets, included patients with ACL injuries, patients with OA, (a) the 464 3.0 T MRI T1p-weighted (b) 174 3D double-echo steady-state (DESS) that were taken from OAI. The datasets were divided into training, validation, and time-point testing into the ratio of 70:20:10. The automatic segmentation performance was calculated by dice coefficient scores for both datasets of six knee MR parts FC, LTC, MTC, PC, LM, and MM. The DSC range has remained 0.632 to 0.699 only for the case of T1p-weighted on validation data with the processing time of 2.5 s. In the case of DESS, the DSC range was higher at 0.878 to 0.753 with the lower computational time of 8 s. The study’s limitation was a lack of ground truth, and that it did not segment more parts of the meniscus and cartilages.

Flannery et al.’s [55] study was an automated segmentation method MR imaging data total of 246 sagittal Constructive Interference in Steady State (CISS) scans BEAR I and BEAR II of intact ACLs. The U-net CNN architecture was a configured segmentation by symmetric down-sampling and depth 5, kernel size 5 × 5, and batch normalization. The model’s performance was evaluated on 29 samples through Dice coefficient, precision, and sensitivity of 0.84, 0.82, and 0.85, respectively. The limitations of the study were that the U-Net model was not trained on low-resolution MRI sequences and other sequences. Furthermore, the model has not used transfer learning to segment ACL reconstruction and repairs.

Flannery et al.’s [56] study was an automatic segmentation quantitatively of reconstructed anterior cruciate ligament and graft after surgery MRIs. The U-Net 2D CNN transfer learning approach was used on the large dataset named Bridge Enhanced ACL Repair (Bear I and Bear II) [57,58,59] of 76 subjects and 45 ACLR issues. Firstly, the base U-Net model was trained on 3480 sagittal slices out of 4920 for segmenting intact ACL and evaluated results through Dice coefficient, sensitivity, and precision of 0.84, 0.85 and 0.82, respectively. Secondly, the transfer learning approach, frozen four layers on BEAR Dataset, performed a lower dice coefficient, sensitivity, and precision of 0.80, 0.82, and 0.79. Thirdly, the ACL graft segmentation results were 0.78, 0.80, and 0.78 on the same evaluation metrics after 200 sagittal slices for testing out of 2400. Besides performance gain after applying transfer learning in both ACL repair and graft segmentation cases, this study was limited only to a MR CISS sequence on a single make scanner.

Almajalid et al.’s [60] used a customized U-Net model to identify the knee bone segmentation of tibia, femur, and patella bones. The Imorphics dataset OAI of 99 knee MRI cases with 160 2D slices was used. The study was trained on only 69 patients, a small dataset with modification of U-net with adam optimizer and softmax activation function. The segmentation result achieved a dice coefficient of 96.94% and a similarity index of 93.98% on testing 15 cases. The study has the limitation that data labeling was time-consuming for segmentation tasks and evaluated on MRI DESS sequences only.

In this section, some of the research studies are introduced that were conducted to segment knee various parts using deep learning. It is clear from the above literature that the accuracy, similarity, and dice coefficient score obtained in individual research work is not currently satisfactory. Most of the studies state the limited number of samples for segmentation and lack of ACL tear segmentation.

## 3. Materials and Methods

This section presents the methods and materials used in this study. Section 3.1 is about the three classes datasets of MR images description used in the proposed method. The proposed CNN is presented in Section 3.2.

### 3.1. DataSet

The original database used in this study was acquired from the knee 917 ACL samples of MRI Stajduhar et al. [61], sponsored by the clinical hospital center in Rijeka, Croatia. The patient age and gender was anonymous in the dataset. The three classes consisted of 690 healthy, 172 normal and 55 complete rupture tears of MR images. The original image size was 330 × 330 × 32 respectively, in width, height and depth. Each slice ACL was manually segmented and served as the ground truth.

### 3.2. Proposed Segmentation Framework

Our proposed segmentation framework is composed of three main phases as follows:

- Phase 1: The pickle ACL MR images were converted into JPEG using an algorithm described in Section 3.2.1.
- Phase 2: The knee mask or ground truth images generation and Javascript object notations (JSON) file creation process were explained in Section 3.2.2.
- Phase 3: Section 3.2.3 was explained with the proposed model based on U-Net CNN.

**Figure 3:**
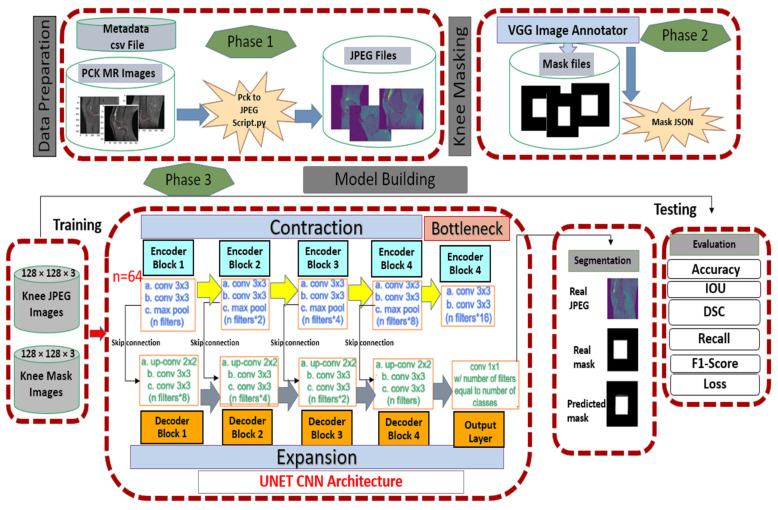
The segmentation framework of our proposed approach with phase 1 of data preparation, phase 2 of knee mask generation and phase 3 of model building with training and testing on our U-Net CNN architecture.

The detailed framework of our segmentation is shown in Figure 3.

#### 3.2.1. Data Preparation Conversion of JPEG Images

The dataset is in pickle format and such format is encrypted. The short name of the pickle file, which is encountered while reading its format. A pickle file is a unique format. It can hold a minimum of 8 images and a maximum of 32 shots. The method of decrypting pickle files is called un-pickling, so it is used for decryption techniques. After un-pickling images, they are converted into grayscale JPEG format. The process of turning pickle files into JPEG files by python script of pickling algorithm is described in Table 1.

#### 3.2.2. Data Preparation Conversion of Knee Masking

**Figure 4:**
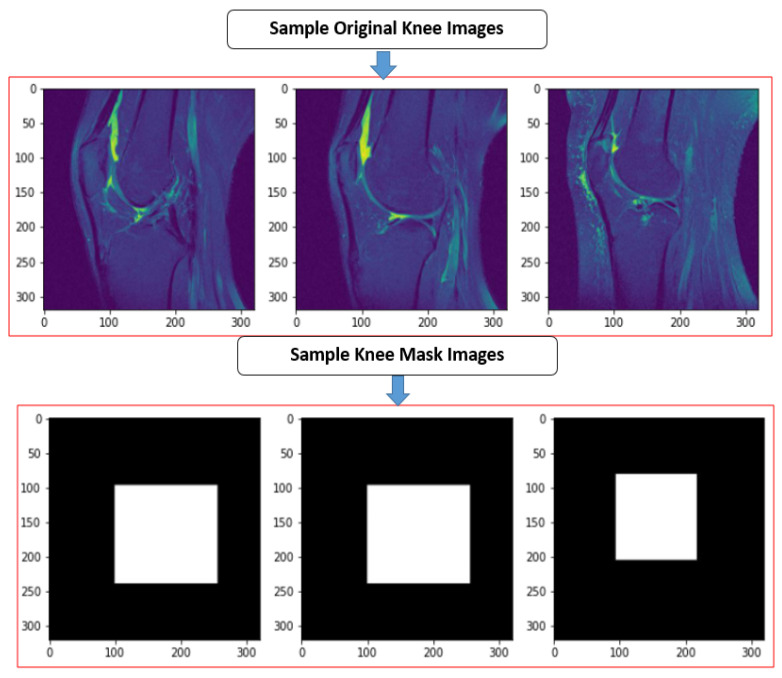
The sample true JPEG images and their knee mask images.

After unpickling all the pickle files into JPEG, we have to label the images into masking with the classes given in the CSV metafile. For this, each JPEG image was loaded into VGG image annotator (VIA) standalone manual annotation software [62]. For labeling the ROI area, the ROI script file was used to identify which particular image contained the data. The VIA tool marked out the labeling box of the JPEG. It created the knee mask and allotted their class labels into JSON (JavaScript Object Notation) format file. The sample jpeg images and knee mask images dimensions are 320 × 320 × 3, shown in Figure 4. The total number of knee JPEG and knee mask images was 15,268 slices.

#### 3.2.3. Our U-Net Convolutional Neural Network Architecture

U-Net is the auto-encoder based is convolutional neural architecture for fast and precise segmentation of images. The Architecture consists of a contracting path and an expensive path [26]. The U-Net combines the context information from the downsampling path with the localization information. There is no dense layer, so images of different sizes can be used as input. The use of massive data augmentation is important in domain-like biomedical segmentation. The three main stages are explained below:

- Contracting/downsampling path

The contracting path is composed of 4 blocks. Each block is composed of two blocks of 3 × 3 convolutional. After this, convolutional 2 × 2 max pooling is applied.

- Bottleneck

There is a bottleneck layer between the contracting and expanding path. The bottleneck layer is simply 2 convolutional layers.

- Expanding/ upsampling path

The expanding layer is the decoder layer. The expanding path is also composed of 4 blocks. Each of these blocks is taken of deconvolutional layer with stride 2. The corresponding cropped feature map is concatenated from the contracting path. At the end, again two blocks of 3 × 3 convolutional, Relu activation function with batch normalization are used.

**Figure 5:**
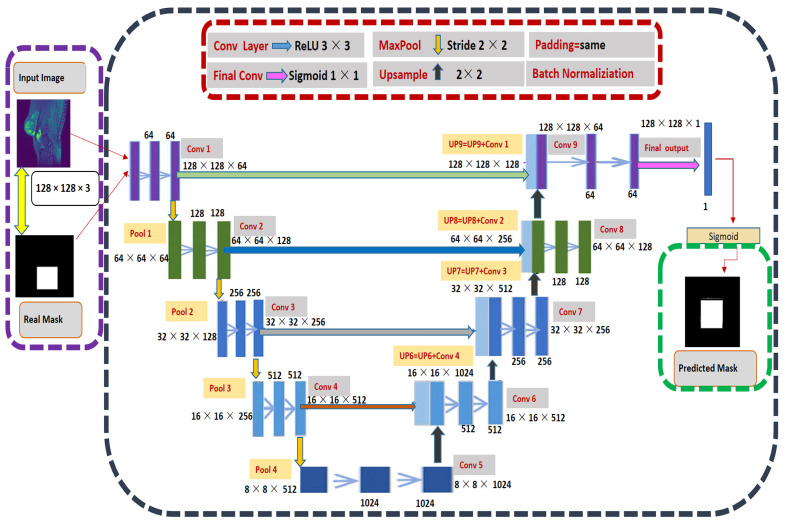
The complete process of modified U-Net Model with eight convolutional layers, four maximum pooling and upsample layers training and prediction.

All of the JPEG and knee mask images were resized into 128 × 128 × 3 before being entered into the input layer, and the filters were fed 64, 128, 256, 512 and 1024, respectively. The first step of two convolution layers (Conv 1) output and kernel size was 128 × 128 × 64, 3 × 3, respectively, with rectified linear unit (ReLU). The padding same represent extra pixels on the edges and output image is the same as input. The next step was the max pooling with stride 2 × 2, the dimensions become half 64 × 64 × 64 of 128 by 128 as used 2 × 2 kernel. The two convolutional layers (Conv 2) output dimensions 64 × 64 × 128 where 128 filter. Repeat this process until bottleneck sage. At this stage, the pooling layer (Pool 4) dimension was 8 × 8 × 512, and on the right side 8 × 8 × 1024. The upsampling (UP6) again used stride 2 × 2 generated the dimension of 16 × 16 × 1024 after concatenating. Then couple of convolutional layers, and the result was 16 × 16 × 152. The batch normalization was used act as a regularizer, eliminating the need for Dropout [63]. Repeat this process until final layer of convolution, which was 128 × 128 × 1. Figure 5 illustrates the complete step by step approach of our U-Net.

At the end, we adjusted the hyper-parameter while compiling the model. We used optimizer adaptive moment estimation (Adam) [64] instead of stochastic gradient descent optimizer and changed the activation function from softmax to sigmoid [65]. The sigmoid function is defined in Equation (1).

(1) $$y=\frac{1}{1+e^{-x}}$$

The other adjustment is loss function in the metrics, we used binary cross entropy and dice loss (BCE). This loss combines Dice loss with the standard binary cross-entropy (BCE) loss that is generally the default for segmentation models [66].

The hyper-parameters adjustment details with their values are described in Table 2.

## 4. Experimental Results

In this section we described the experiment setup, splitting of data and evaluation metrics.

### 4.1. Experimental Setup

The experiments were carried out on Google Colab with Python version 3.8.8. The processor was Intel(R) Xeon(R) Silver 4110 CPU 8 cores with 16 GB RAM. The execution is totally based on CPUs rather than on GPU. The research architectures have been implemented using Keras implementation in Tensorflow 2.7.0. Moreover, Jupyter Notebook has been used in Anaconda on Windows 10 Operating System.

### 4.2. Train/Test Split

To prepare the data for our model, we randomly divided the 75% cases into training, and 25% test sets for both cases of JPEG and mask knee slices. The details of ratio of the samples are described in Table 3.

### 4.3. Evaluation Metrics

The segmentation tasks need to output an image the same size as the input image, and the network architecture needs to be adapted accordingly with this output. The value of each pixel or voxel represents the segmentation class of that voxel. The output segmentation is measured against a ground truth often an expert delineation using the metrics. In segmentation, we want to classify whether it is background or foreground. The foreground pixels belonged to the class. We calculated score through accuracy, Intersection over union (IoU), Dice similarity coefficient (DSC), Precision, Recall, and F1-Score. Furthermore, the loss value we also evaluated through Binary Cross Entropy and Dice Loss. The explanation of evaluations metrics is described below:

1. Accuracy

The average accuracy of the model is calculated by the fraction of total area of foreground plus background by the foreground. The accuracy Equation (2) is as below.

(2) $$Accuacy=\frac{Foreground\;area\;of\;knee\;Mask}{Total\;area\;of\;foreground+Total\;background\;area}$$

2. Intersection over Union

The intersection is the area of the intersection between label (A) and the prediction (B), and it should be maximizing (True Positive). The union should be minimizing because some part of the union is outside the label, in which occur errors (False Positive and False Negative). The IoU Equation (3) is as below.

(3) $$IOU=\frac{Intersection\left(A\cap B\right)\;}{Unioin\;\left(A\;\cup \;B\right)}$$

3. Dice Coefficient

It is two times the intersection divided by the sum of the sizes of two segmentation regions. Both these metrics are zero when there is no overlapping between the predicted and ground truth regions and one when there is perfect overlap. The dice coefficient score Equation (4) is as below.

(4) $$Dice\;Coefficient=\frac{\;2\times Intersection\left(A\cap B\right)\;}{Unioin\;\left(A\;\cup \;B\right)+Intersection\left(A\cap B\right)}\;$$

4. Precision

The fraction of corrected segmented positive images by a total number of true positive and number of false positive. The precision Equation (5) is as below.

(5) $$Precison=\frac{Total\;number\;of\;corrected\;prediction\;\left(TP\right)}{TP+FP}\;$$

5. Recall

The fraction of all positive images in three classes is correctly predicted as positive by the classifier. The recall Equation (6) is as below.

(6) $$Recall=\frac{\left(TP\right)}{\;\;TP+FN}\;$$

6. F1 score

It combines precision and recalls through harmonic means. The F1 score Equation (7) is as below.

(7) $$F1\;Score=\frac{1}{\frac{1}{p_{\mathrm{recision}}}+\frac{1}{\mathrm{recall}}}$$

**Figure 6:**
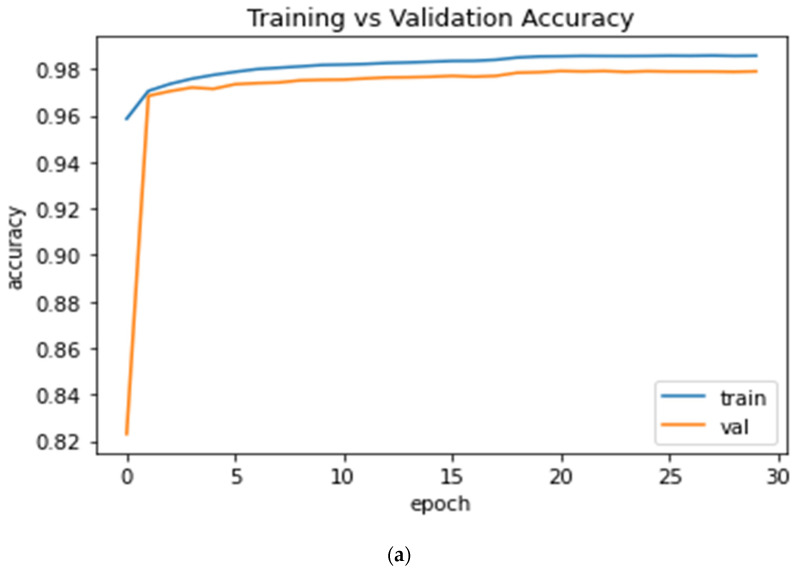

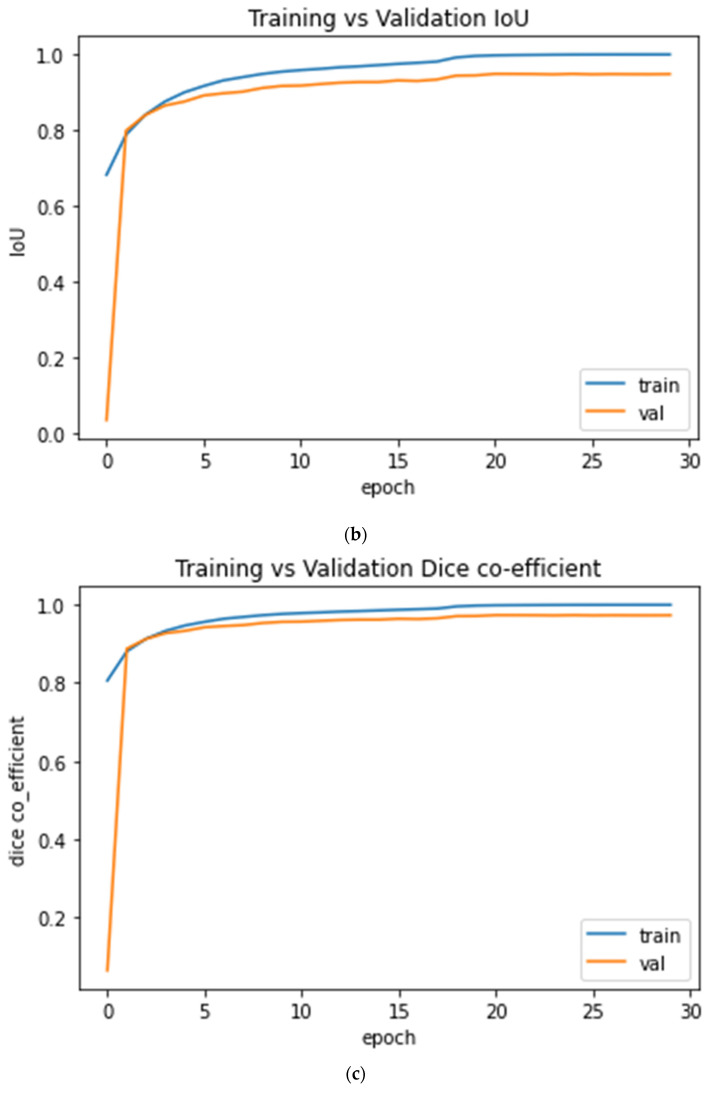

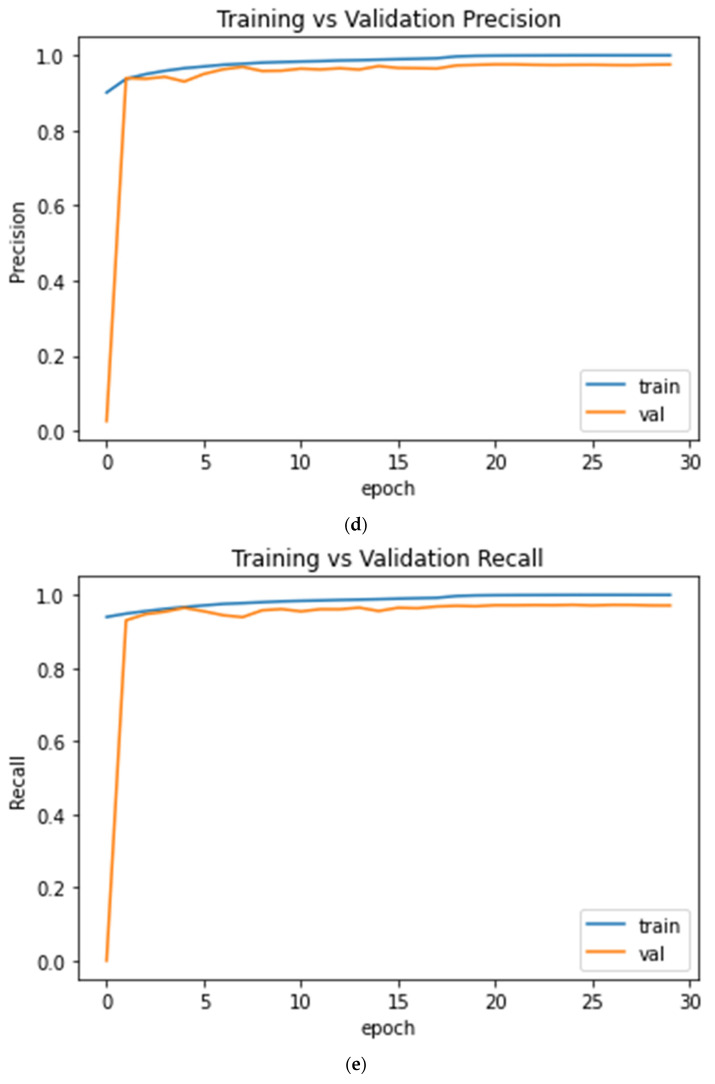

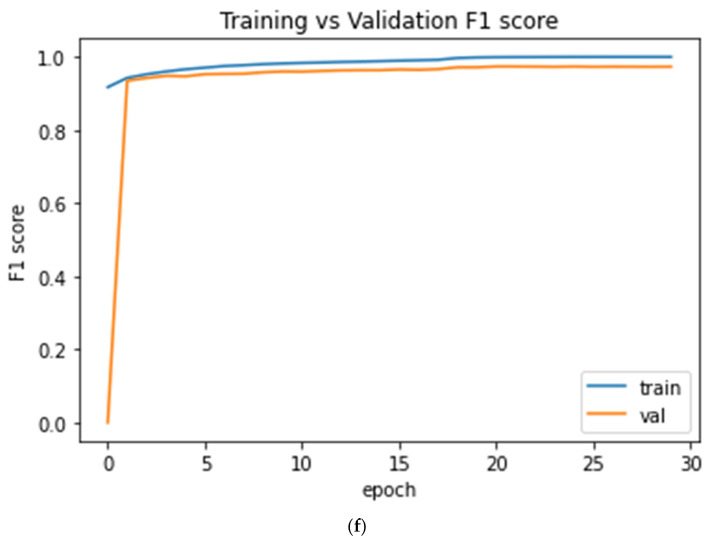
The evaluation score after 30 epochs training vs. validation plots; (**a**) Accuracy score curve; (**b**) IoU score curve; (**c**) dice_coeff score curve; (**d**) Precision score curve; (**e**) recall score curve; and (**f**) F1 score curve.

Figure 6 further shows the curves of training vs. testing accuracy, Iou, dice_coeff, precision, recall and F1 score.

7. Binary Cross Entropy Dice Loss (BCE-Dice Loss)

It is widely used for classification objective, and as segmentation is pixel level classification, it works well. This loss combines Dice loss with the standard binary cross-entropy (BCE) loss that is generally the default for segmentation models. Combining the two methods allows for some diversity in the loss, while benefitting for Binary Cross Entropy and Dice Loss between a true and a predicted classification. The Equation (8) for Binary cross entropy with dice coefficient is as below.

(8) $$BCE\;loss\left(A,B\right)=-1/N\;\overset{N}{\underset{i=1}{\sum }}(A\;log\left(B\right)+\left(1-A\right)\;\;log\left(1-B\right))$$

8. Dice Similarity Loss (DSC)

The Dice coefficient is a widely used metric for the imbalance category to calculate the similarity between two images. If the DSC loss will have a negative impact on the back propagation. The formula of dice is as below in Equation (9)

(9) $$\mathrm{DiceLoss}=1-2\frac{\sum_{i=1\;}^{N\;}A_{i}{\;B}_{i}}{\sum_{i=1}^{N}A_{i}{}^{2}+\;\sum_{i=1}^{N}B_{i}{}^{2}}$$

**Figure 7:**
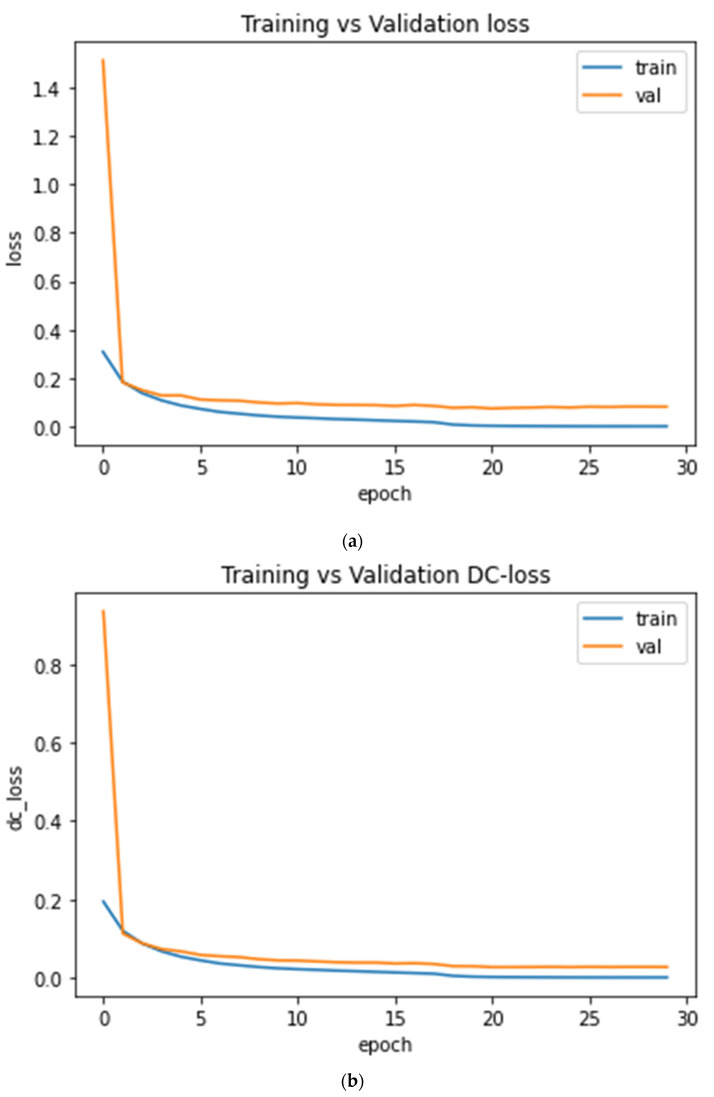
The loss value after 30 epochs training vs. test curves; (**a**) BCE-Dice Loss curve; and (**b**) Dice Similarity Loss curve.

Figure 7 shows the plot of training and validation loss of BCE-Dice loss and dice loss after 30 epochs.

## 5. Discussion

**Figure 8:**
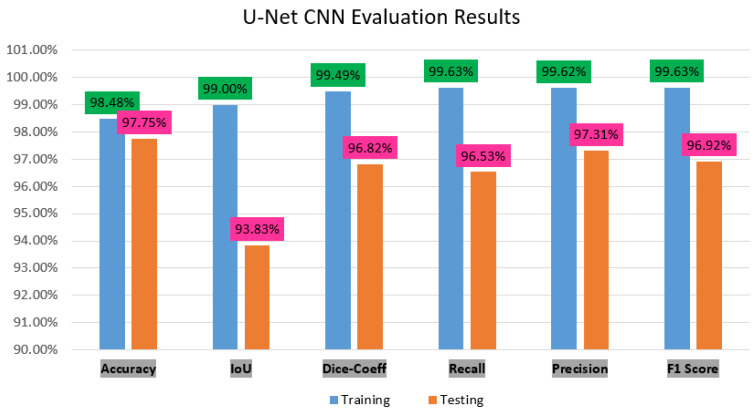
The performance charts of accuracy, IoU, dice_coeff, recall, precision and F1 score.

In this study, we focused on the semantic segmentation deep learning approach of U-Net CNN architecture to segment the ACL MR images automatically. The ACL segmentation is difficult in the case of pickle images, for this we prepared these images into mask images and then segment it. The experiment results are shown in Figure 8 after evaluation of modification in the U-Net model.

Our results demonstrate in the figure above through deep learning there is not much difference in the accuracy score between the training and testing, respectively, 98.48% and 97.75%. However, the IoU, dice, coeff, revall, precision and F1 score difference was between 5% to 3%. Therefore, our model performance score has shown very good results.

**Figure 9:**
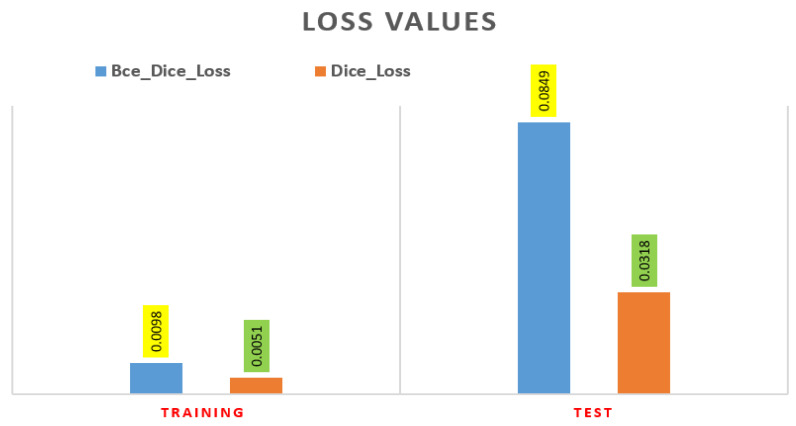
The Error Loss of Dice Loss and BCE_Dice-Loss on training and test dataset.

Furthermore, the error loss between the training and test are shown in Figure 9. The test error dice loss is much less as compared with BCE-dice-Loss, respectively, 0.00318 and 0.0849.

**Figure 10:**
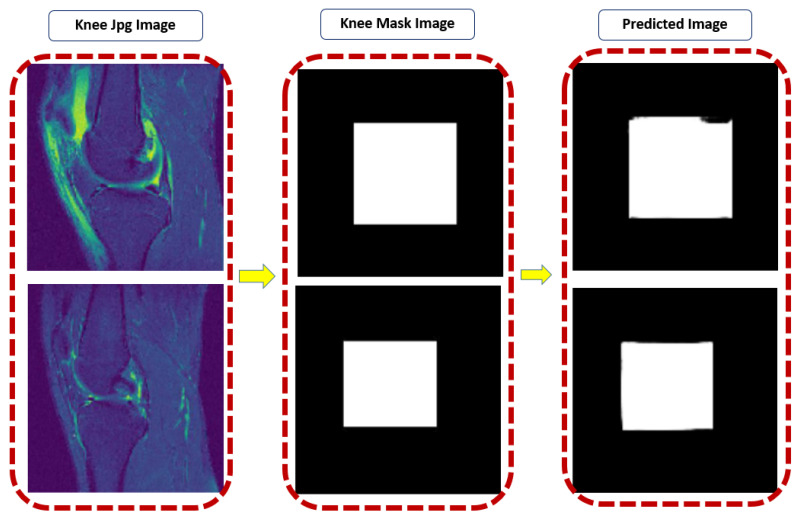
After testing actual knee mask and predicted mask.

Figure 10 shows our predicted images after segmentation of some samples after testing with true images.

Table 4 describes the state-of-art work comparisons with our segmentation model, and evaluation metrics.

The above Table 4 clearly shows that our result of segmentation was performed with good results.

There are five studies in Table 4 applying CNN model without U-Net architecture but having problems: limited dataset of training voxels, not use stage layer to the pre-trained CNN model [31], manual segmentation was time-consuming [33], less accuracy was found even in cases of small structures of knee [35], processing time took much time for segmentation automatically [39], and not investigated multitask learning for segmentation and registration to decease model size [43].

There are six studies that apply U-Net CNN architecture but have limitations: less performance, absence of ground truth [47], lack of image annotation uncertainty [49], lack of gold standard [50], segmentation is performed on only a few parts of knee [50], low resolution of MR images [51], limited only for MR CISS sequences in a single make scanner [52] and data labeling is time consuming [56].

There arethree limitations of our study. Firstly, the data labeling is time-consuming because the bounding box was generated through a VGG annotator tool. Secondly, the segmentation algorithm is also time-consuming to train our U-Net model. Thirdly, the dataset was limited only to ACL tear segmentation.

## 6. Conclusions

The semantic segmentation of medical images is a more advanced and complicated task. A fully automated modified U-Net convolutional neural network method was applied and segmented the ACL tear of MR images. From the public knee MRI dataset, 917 volumes and 15,268 3D knee MRI slices have been used in this study after converting knee masking. It is the first attempt of dataset preparation into JPEG and knee masking. Without any human intervention, the trained system takes the 11451 MRI slices as input and predicts outputs of the segmentation. In the experiments, the intersection over union, dice coefficient, precision, recall and F1 score was 93.83%, 96.82%, 97.31%, 96.53% and 96.92%, respectively, on the testing dataset for whole knee ACL segmentation. We further conducted binary cross entropy combined with dice coefficient loss and dice loss on training and test datasets. These losses were 0.0849 and 0.0318 on test data after 30 epochs. In addition, we compared the propose model with other state-of-art of all knee components and models. It clearly shown that our result was very promising as compared to other studies. In the future, we could apply classification models on predicted knee images. The instant segmentation can also be applied on knee mask images.

## Figures and Tables

**Table 1:** The algorithm of turning pickles into JPEG images.

**Input: Load all pickle files into data**
for id in enumerate(data):
reshape each d into image till last image
save each image into JPEG form
**Output: show all images**

**Table 2:** The hyper-parameters adjustment and values.

Hyper-Parameters Adjustment	Value
Input Image	128 × 128 × 3
Batch Size	32
Number of Epochs	30
Learning rate	0.0001
Optimizers	Adam
Loss Function	Binary cross-entropy and Dice loss

**Table 3:** The training and test split ratio of samples of knee MR images.

Table 11451	Knee JPEG Images	Knee Mask Images
Training Data	11451	11451
Test Data	3817	3817

**Table 4:** The segmentation of various knee comparisons with our ACL segmentation.

Author, Year	Technique/Model	Segment Part ACL Yes/NO	Segment Part	Evaluation				
				DSC	IoU	Recall	Precision	F1-Score
Prasoon [34], 2013	2D CNN	No	TC	0.824	-	0.819	-	-
Deniz, Xiang, Hallyburton, Welbeck, Babb, Honig, Cho and Chang [35] P, 2018	3D CNN U-Net	No	PF	0.950	-	0.950	0.950	-
Zhou, Zhao, Kijowski and Liu [36], 2018	CNNVGG16	No	TF, muscle, non-spec tissue	0.910	-	-	-	-
Ambellan, Tack, Ehlke and Zachow [38], 2019	CNN	No, OAI Imorphics	FC	0.894	-	-	-	-
			MTC	0.861	-	-	-	-
			LTC	0.904	-	-	-	-
Xu and Niethammer [42], 2019	CNN	No	Bone	0.968	-	-	-	-
			Cartilages	0.776	-	-	-	-
			knee part other	0.872	-	-	-	-
Burton, Myers and Rullkoetter [43], 2020	U-Net	No	Femur, FC, TC, PC, Tibia, petella	0.989	0.971	-	-	-
Liu, Zhou, Samsonov, Blankenbaker, Larison, Kanarek, Lian, Kambhampati and Kijowski [46], 2018	2D CNN	No	Femur	0.96	-	-	-	-
			Tibia	0.95	-	-	-	-
			FC	0.81	-	-	-	-
			TC	0.82	-	-	-	-
Tack, Mukhopadhyay and Zachow [49], 2018	U-Net	No	LM	0.889	-	-	-	-
			MM	0.838	-	-	-	-
Raj [51], 2018	U-Net	No	OAI: FC	0.849	-	-	-	-
			LM	0.849	-	-	-	-
			LTC	0.856	-	-	-	-
			MM	0.801	-	-	-	-
			MTC	0.806	-	-	-	-
			PC	0.784	-	-	-	-
			SK110:FC	0.834	-	-	-	-
			TC	0.825	-	-	-	-
Pedoia, Norman, Mehany, Bucknor, Link and Majumdar [53], 2019	U-Net	No	Meniscus	-	-	0.899	-	-
			Cartilage	-	-	0.801	-	-
			Normal lesion	-	-	0.807	-	-
Norman, Pedoia and Majumdar [54], 2018	U-Net	No	FC	0.878	-	-	-	-
			LTC	0.820	-	-	-	-
			MTC	0.795	-	-	-	-
			PC	0.767	-	-	-	-
			LM	0.809	-	-	-	-
			MM	0.753	-	-	-	-
Flannery, Kiapour, Edgar, Murray and Fleming [55], 2021	U-Net	Yes repair BEAR	ACL	0.840	-	0.850	0.821	-
Flannery, Kiapour, Edgar, Murray, Beveridge and Fleming [56], 2021	U-Net	Yes	ACL Intact BEAR	0.840	-	0.850	0.820	-
			ACL graft	0.780	-	0.801	0.781	-
Almajalid, Zhang and Shan [60]	U Net	No Imorphics OAI	Tibia	0.963	-	0.995	0.988	-
			Femur	0.979	-	0.996	0.988	-
			petella	0.928	-	0.971	0.992	-

Dice Similarity Coefficient= DSC, Femur Cartilage = FC, Tibial Cartilage = TC, Proximal Femur = PF, Lateral Meniscus = LM, Medical Meniscus = MM, Lateral Tibial Cartilage = LTC, Medial Tibial Cartilage = MTC.

## Data Availability

We are using this dataset kneeMRI dataset available online: http://www.riteh.uniri.hr/~istajduh/projects/KneeMRI/ (accessed on: 1 March 2017) in our work from Clinical Hospital Centre Rijeka, under reference [61].
